# Iron deficiency without anemia in children with newly diagnosed celiac disease: 1-year follow-up of ferritin levels, with and without iron supplementation

**DOI:** 10.1007/s00431-024-05721-1

**Published:** 2024-08-27

**Authors:** Tal Ben-Ami, Anna Trotskovsky, Chani Topf-Olivestone, Michal Kori

**Affiliations:** 1 Department of Pediatrics, Kaplan Medical Center, Rehovot, Israel; 2 Pediatric Hematology-Oncology Unit, Kaplan Medical Center, Pasternak St, Rehovot, Israel; 3 Pediatric Gastroenterology, Assuta Medical Center, Ashdod, Israel; 4 Faculty of Medicine, Hebrew University of Jerusalem, Jerusalem, Israel; 5 Pediatric Gastroenterology, Kaplan Medical Center, Rehovot, Israel

**Keywords:** Iron deficiency, Celiac disease, Iron supplement

## Abstract

Iron deficiency (ID) without anemia is common in children with newly diagnosed celiac disease (CD). We aimed to assess the effect of iron supplementation versus no treatment on ferritin levels in newly diagnosed CD patients with ID adhering to a gluten-free diet (GFD). A retrospective review of children < 18 years, with low ferritin (≤ 10 ng/mL) and normal hemoglobin levels diagnosed between 12.2018 and 12.2021. We compared hemoglobin and ferritin levels between patients who received supplemental iron to those who did not. Data, including demographics, laboratory tests, and anthropometrics, were collected at baseline, and at 6 and 12 months following the initiation of the GFD. Adherence to GFD was assessed at each visit. Among 304 children diagnosed during the study period, 43 (14.1%) had iron deficiency anemia and 60 (19.7%) ID without anemia. Among children with ID, 29 (48%) were female, mean age 7.3 ± 3.9 years. Twenty-nine (48%) children received iron supplementation, and 31 (52%) did not. At the 12-month follow-up visit, tissue transglutaminase levels decreased significantly (*p* < 0.001), from a mean baseline level of 226.6 ± 47.8 to 34.5 ± 46 U/mL in children that received iron supplementation and from 234.2 ± 52.4 to 74.5 ± 88.7 U/mL in non-treated children, with no significant difference between the groups *p* = 0.22. Ferritin levels increased significantly (*p* < 0.001), from 9.0 ± 4.7 to 25.2 ± 20.8 ng/mL in patients who received supplementation and from 8.9 ± 3.8 to18.6 ± 9.5 ng/mL in patients who did not, with no significant difference between the groups (*p* = 0.46).

*Conclusion*: Most children with newly diagnosed celiac disease and iron deficiency, who adhere to GFD, will normalize ferritin levels within 12 months without the need of iron supplementation.

**Table:** 

**What is Known:** *• Iron deficiency and iron deficiency anemia are common in newly diagnosed celiac disease.* *• Improved iron absorption may follow mucosal healing process in patients adhering to a strict gluten-free diet.*	
**What is New:** *• This single-center, retrospective cohort study evaluated the effect of iron supplementation versus no treatment on ferritin levels in children with newly diagnosed celiac disease with iron deficiency adhering to a gluten-free diet.* *• Most children with newly diagnosed celiac disease and iron deficiency, who adhere to gluten-free diet, will normalize ferritin levels within 12 months without the need of iron supplementation.*

**What is Known:**

*• Iron deficiency and iron deficiency anemia are common in newly diagnosed celiac disease.*

*• Improved iron absorption may follow mucosal healing process in patients adhering to a strict gluten-free diet.*

**What is New:**

*• This single-center, retrospective cohort study evaluated the effect of iron supplementation versus no treatment on ferritin levels in children with newly diagnosed celiac disease with iron deficiency adhering to a gluten-free diet.*

*• Most children with newly diagnosed celiac disease and iron deficiency, who adhere to gluten-free diet, will normalize ferritin levels within 12 months without the need of iron supplementation.*

## Introduction

Celiac disease (CD) is an immune-mediated enteropathy triggered by exposure to dietary gluten in genetically predisposed individuals. The world prevalence of CD is estimated at 1% [1, 2]. Iron deficiency anemia (IDA) and iron deficiency (ID) without anemia are among the most common extra-intestinal manifestations of CD, and in some cases, they are the main or even the only clinical manifestation of the disease [3]. The prevalence of anemia in newly diagnosed CD patients varies from 25% to up to 82%, depending on the study in question, including studies in adult patients [4, 5]. Sanseviero et al. identified 112 (21.6%) children with IDA and 115 (22.2%) with ID, in a cohort of 518 newly diagnosed pediatric CD patients, confirming that iron depletion and IDA represent a frequent finding at the time of diagnosis of pediatric CD [6]. In a Finnish pediatric study, 42.5% of 197 newly diagnosed CD patients had ID [7].

Iron deficiency (ID), with or without anemia, in celiac CD can be explained by multiple etiologies. The primary mechanism is malabsorption attributable to small intestinal mucosal damage, particularly villous atrophy [4, 5]. Concurrently, reduced iron intake, often secondary to abdominal discomfort and diarrhea, and occult gastrointestinal hemorrhage are contributory factors. Additionally, celiac disease can precipitate deficiencies in other micronutrients and vitamins, notably folic acid and vitamin D [8].

A strict gluten-free diet (GFD) remains the sole therapeutic intervention for CD. Upon gluten exclusion, there is a decrease in celiac-specific antibodies, including tissue transglutaminase (TTG) antibodies, which typically normalize concomitantly with the restoration of the intestinal mucosa [9]. This mucosal healing process may take up to a year or more. Iron absorption is enhanced as healing of the intestinal mucosa is established [9].

The routine management of children with newly diagnosed CD includes the evaluation of hemoglobin and iron stores measured by ferritin levels, at diagnosis and during follow-up. Hemoglobin and ferritin levels are assessed according to age and gender [10, 11]. The management of IDA and ID in CD is mainly focused on iron repletion [12]. In patients with mild to moderate IDA, oral iron replacement in parallel to the GFD is the mainstay of treatment. Children with severe iron deficiency at presentation may require intravenous iron [13]. The GFD alone may improve mild forms of IDA or ID in patients with CD; however, the approach and management in these cases is not well established [14–16]. Our primary aim was to assess the effect of iron supplementation versus no treatment on ferritin levels in newly diagnosed CD patients adhering to a GFD.

## Methods

A retrospective review of medical charts of children < 18 years, diagnosed with CD, between December 2018 and December 2021. CD was diagnosed with or without a biopsy based on the latest ESPGHAN criteria [2]. Among patients with newly diagnosed CD, we identified children with iron deficiency anemia and with iron deficiency without anemia based on normal hemoglobin levels for age and gender, as well as ferritin levels of ≤ 10 ng/mL [10, 11]. All patients began a strict gluten-free diet (GFD) following their diagnosis. Iron supplementation was administered to all children diagnosed with iron deficiency anemia (IDA) at time of CD diagnosis. Iron supplements were not prescribed to patients with isolated iron deficiency (ID) without anemia. Nevertheless, about half of the patients with ID were advised by their pediatrician to start iron supplementation at the time of CD diagnosis. The subsequent analysis focused on the 60 children with ID without anemia from our cohort. Baseline data collected included demographic information, laboratory results (hemoglobin (Hb), ferritin, and TTG levels), and anthropometric measurements. Patients with IgA deficiency were excluded. TTG testing was performed in all patients by the automated IgA-TTG analyzer (Bioplex 2200) as previously described [17]. Normal TTG levels are < 15 (U/mL), and maximal levels are presented as above > 250 (µ/mL). Follow-up data included Hb, ferritin, and TTG levels at 6 and 12 months following the initiation of GFD. Data on adherence to GFD was assessed using medical records documentation. Data on initiation and adherence to iron supplementation suggested by the child’s pediatrician were assessed using medical record documentation, digitalized pharmacy purchases, and telephone interviews.

The study was performed in line with the Declaration of Helsinki and was approved by Kaplan Medical Centers’ ethics committee.

### Statistics

Categorical and nominal variables were reported by prevalence and percentages; they were analyzed by Pearson’s chi-square (*χ*^2^) test. Continuous variables were presented as means ± standard deviations and were tested for normality by Kolmogorov-Smirnova test, and when abnormal distribution was found, non-parametric tests were performed. A Spearman correlation was performed between the differences of the measurements for the parameters (weight percentiles, ferritin, hemoglobin, and TTG). *P* value < 0.05 was considered statistically significant data were analyzed using SPSS.

## Results

Among 304 children diagnosed with CD during the study period, we identified 43 (14.1%) children with iron deficiency anemia and 60 (19.7%) with ID without anemia. Among the 60 children with ID, which are the cohort of the current study, 29 (49%) were females with a mean age of 7.3 ± 3.9 years, 10 were diagnosed with the no-biopsy approach, and 50 with endoscopy and biopsy. All children started a GFD post diagnosis. The iron supplementation group included 29 (48%) children who received oral iron supplementation at diagnosis, based on primary physician recommendation. The non-treated group included31 (52%) children that did not receive any iron treatment. The baseline characteristics and laboratory tests of the patients did not differ between the two groups (Table 1).

**Table 1:** Baseline characteristics and laboratory data

		Iron supplementation group (*n* = 29)		No iron supplementation group (*n* = 31)	
		Mean	SD	Mean	SD
Age (years)		7.4	4.3	7.5	3.6
Gender (female)		11 (38%)		18 (58%)	
Weight percentile		33	24	37	30
Height percentile		38	28	34	30
Lab	Hemoglobin (gr/dL)	12.4	0.68	12.7	0.65
	Ferritin (ng/mL)	9.4	5.12	9.9	5.37
	TTG (U/mL)	213.6	63	221.1	61

*TTG* tissue transglutaminase

**Fig. 1:**
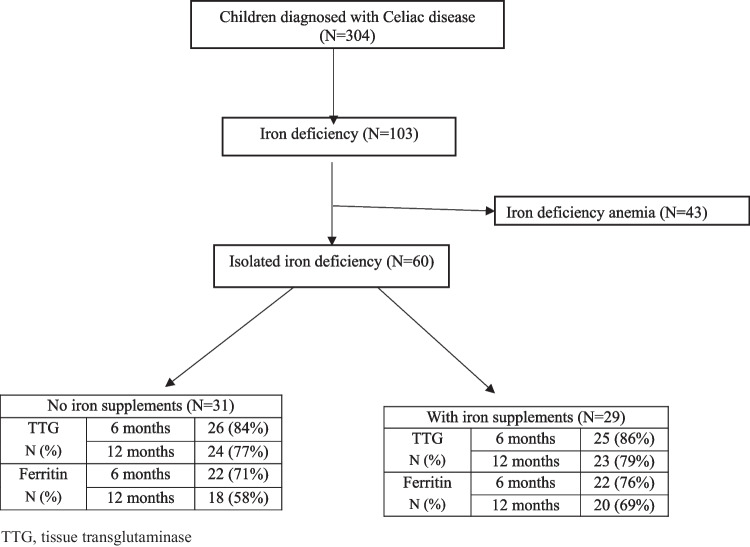
Study population

Follow-up data were systematically collected at 6-month and 12-month intervals following the initiation of the GFD. The dataset included hemoglobin, ferritin, and TTG levels for all patients at least once during the first year of follow-up (Fig. 1). Hemoglobin levels remained within the normal range for all patients throughout the study; thus, these data are not reported here.

Since not all children attended both the 6-month and 12-month visits, the statistical analysis of changes in TTG and ferritin levels was conducted only for patients who had complete data across all time points: 19 and 16 children respectively in the iron-supplementation group and 19 and 11 children in the non-treated group (Table 2).

**Table 2:** TTG and ferritin levels at baseline and follow-up

Timeline	Iron supplementation			No iron supplementation		
	Diagnosis	6 months	12 months	Diagnosis	6 months	12 months
Number of patients	29	25	23	31	26	24
TTG						
Mean (SD)	213.6 (63)	58.3 (69)	35 (42.8)	221.1 (61)	82.6 (83)	64.5 (81.5)
Number of patients	29	22	20	31	22	18
Ferritin						
Mean (SD)	9.4 (5.1)	18.9 (15.9)	23.5 (19.1)	9.9 (5.4)	17.3 (13.6)	16.1 (8.9)

TTG is measured in U/ml (units per milliliter)

*TTG* tissue transglutaminase

**Fig. 2:**
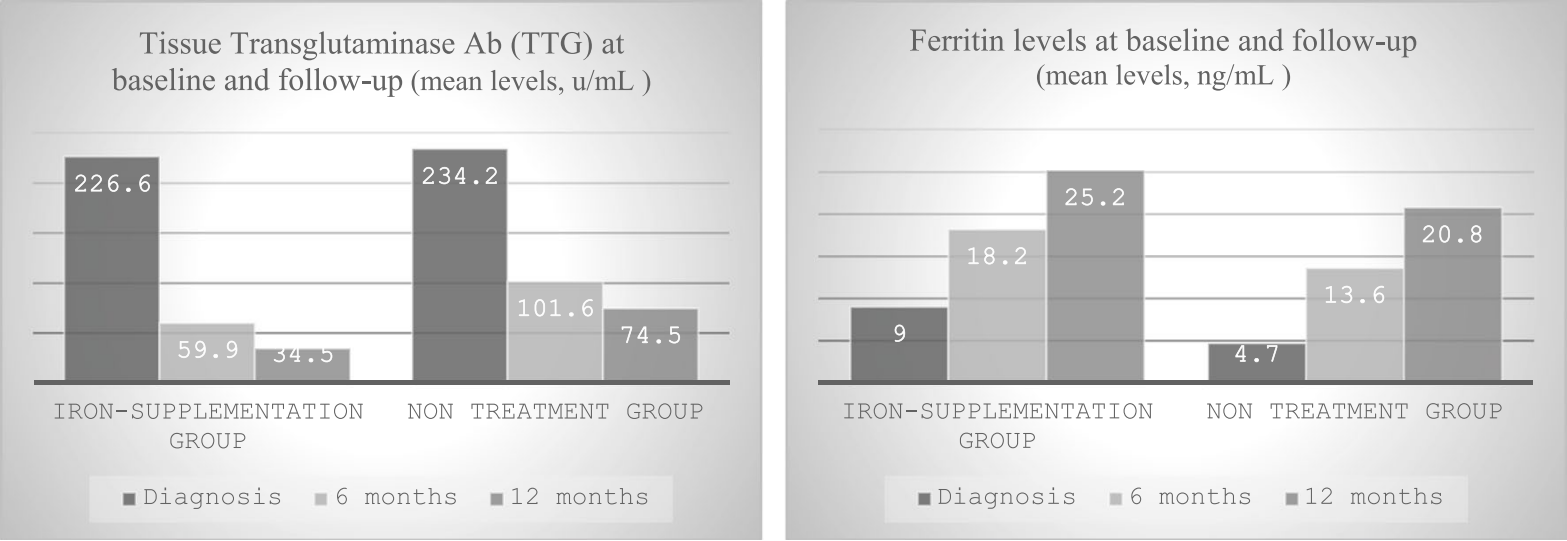
Tissue transglutaminase (TTG) antibody and ferritin levels at baseline, 6-month, and 12-month time points

TTG levels decreased significantly in both groups. In the iron-supplementation group, TTG levels decreased from a mean baseline of 226.6 to 34.5 ± 46 (U/mL), and in the non-treated group from 234.2 ± 52.4 to 74.5 ± 88.7 (U/mL) (*p* < 0.001). There was no significant difference between the two treatment groups (*p* value = 0.22). Ferritin levels increased significantly in both groups. In the iron-supplementation group, ferritin values increased from 9.0 ± 4.7 to 25.2 ± 20.8 (ng/mL), and in the non-treated group, they increased from 8.9 ± 3.8 to 18.6 ± 9.5 (ng/mL) (*p* value < 0.001) with no significant difference between the two treatment groups (*p* value = 0.64) (Fig. 2).

## Discussion

Iron deficiency (ID) and iron deficiency anemia (IDA) are frequent findings in children with newly diagnosed celiac disease (CD) [18–22]. In a large nationwide study from Israel, anemia was significantly more common in adolescents with CD compared to controls, with a hazard ratio of 1.7 (1.5–1.9) [23].

The treatment protocol for newly diagnosed patients with CD mandates adherence to a strict GFD. For pediatric patients presenting with IDA, iron supplementation is recommended as part of their treatment regimen [12, 24].However, evidence is lacking regarding the added benefit of routinely supplementing iron to children with isolated ID. In cases of isolated ID without anemia, our current policy does not include routine iron supplementation, in concordance with the ESPGHAN position paper of follow-up of children with CD [12]. The position paper states that an expectant attitude may be appropriate on the GFD as long as there is improvement in iron stores without supplementation. An increase in iron stores can be expected within 1 year of initiating a GFD, although more evidence based on prospective studies is needed. Poor compliance with the GFD may hamper recovery [22, 25]. Additionally, a diet low in nutritional iron content can predispose children to non-recovery of IDA, especially during growth spurts. The ESPGHAN position paper also notes that the prevalence of subclinical ID is rarely reported during follow-up. In most cases (84–96%), anemia improves or recovers on a GFD [18, 19, 22, 26, 27]. Nonetheless, it is noted that some pediatricians may opt to initiate iron therapy for such patients. Our practice does not actively intervene to contradict these recommendations. This retrospective study evaluates the increase in ferritin levels among newly diagnosed children with CD who were given iron supplements compared to those who were not. Our findings suggest that adherence to a gluten-free diet alone as indicated by the decrease in tissue transglutaminase (TTG) antibody levels can enhance and normalize ferritin levels. Ferritin levels increased significantly after 12 months, both in children who received iron supplementation and in children who did not, with no significant difference between the groups. This could be attributed to the intestinal mucosal recovery in children who maintain a strict GFD, enhancing iron absorption and consequently increasing ferritin levels even in the absence of iron supplementation [14–16].

The gluten-free diet (GFD) is currently the sole effective therapeutic strategy for celiac disease (CD). However, strict compliance with the GFD can be challenging, often resulting in psychological and social difficulties for some patients [9, 28]. Assessing adherence to the GFD in pediatric populations with CD is complex, entailing nutritional evaluations, serological tests, and, in some instances, repeated endoscopic examinations and biopsies [14]. Each of these methods has inherent limitations and may fail to detect lapses in adherence. Additionally, iron supplementation for children with iron deficiency anemia (IDA) is frequently met with suboptimal compliance and can be accompanied by adverse effects, predominantly gastrointestinal in nature [29]. These challenges can further complicate management for patients and their families. Witnessing an increase in ferritin levels without additional iron supplementation may allow clinicians to forego unnecessary iron therapies, offering a clear advantage for patient care.

Studies on the effect of GFD on recovery from IDA are scarce and include mainly adult patients [14, 15, 28, 30]. It has been demonstrated in adult patients that the recovery of anemia usually occurs within 1 year after the commencement of a strict GFD, in most cases, even without additional iron supplementation [30]. In a series of 26 adult with a biopsy-confirmed CD, at 12 months, all but one patient (94.4%) recovered from anemia and 50% from ID [30]. A study conducted by Ståhlberg et al. investigated the impact of (GFD) on iron deficiency in children with celiac disease. The study included 54 children, 30% of whom presented with mild anemia at the time of diagnosis, and 16% had ID. It was found that treatment with a GFD, without iron supplementation, effectively eliminated all signs of iron deficiency and normalized laboratory values in all children [31]. Sansotta et al. conducted a retrospective chart review of pediatric and adult patients with CD, aiming to evaluate the efficacy of the GFD on gastrointestinal and extra-intestinal symptoms including IDA. Out of 554 patients (227 children), 48% of adults and 8% of children had IDA at the time of diagnosis. All the patients started a strict GFD. At the end of the follow-up period (an average of 3.4 years for children and 3.0 years for adults), 85% of adults and almost 100% of children had hemoglobin levels in the normal range [15]. Moreover, a study that evaluated the dietary iron in 132 children with CD adhering to the GFD showed that 84.8% of the participants managed to cover their iron food needs while adhering to a gluten-free diet, indicating that a well-planned gluten-free diet can meet the dietary iron requirements of children with CD [32]. Our study demonstrated that ferritin levels increased significantly after 12 months, in both groups of children with no significant difference between the groups *p* = 0.56, further supporting the practice of refraining from iron supplementation in these children.

The strengths of our study are exemplified by the rigorous follow-up conducted with all newly diagnosed CD patients over the initial 12 months post-diagnosis. However, the study is not without limitations. Its retrospective nature, coupled with incomplete follow-up data for the entire cohort, presents challenges to the conclusiveness of our findings. Most notably, the lack of a standardized protocol for iron supplementation—where the decision was made at the discretion of the child’s pediatrician and the parents—significantly impedes the ability to draw uniform conclusions from the data.

In conclusion, most pediatric patients with newly diagnosed celiac disease who present with iron deficiency appear to normalize their ferritin levels within 12 months by adherence to a gluten-free diet alone, without requiring iron supplementation. To validate these observations, larger-scale, prospective, randomized controlled trials are warranted.

## Data Availability

No datasets were generated or analysed during the current study.

## Abbreviations

CD: Celiac disease
ESPGHAN: European Society for Paediatric Gastroenterology Hepatology and Nutrition
GFD: Gluten-free diet
ID: Iron deficiency
IDA: Iron deficiency anemia
TTG: Tissue transglutaminase

## References

[1] Husby S, Koletzko S, Korponay-Szabo IR, Mearin ML, Phillips A, Shamir R, Troncone R, Giersiepen K, Branski D, Catassi C et al (2012) European Society for Pediatric Gastroenterology, Hepatology, and Nutrition guidelines for the diagnosis of coeliac disease. J Pediatr Gastroenterol Nutr 54(1):136–160 22197856 10.1097/MPG.0b013e31821a23d0

[2] Husby S, Koletzko S, Korponay-Szabo I, Kurppa K, Mearin ML, Ribes-Koninckx C, Shamir R, Troncone R, Auricchio R, Castillejo G et al (2020) European Society Paediatric Gastroenterology, Hepatology and Nutrition guidelines for diagnosing coeliac disease 2020. J Pediatr Gastroenterol Nutr 70(1):141–156 31568151 10.1097/MPG.0000000000002497

[3] Souroujon M, Ashkenazi A, Lupo M, Levin S, Hegesh E (1982) serum ferritin levels in celiac disease. Am J Clin Pathol 77(1):82–86 7055099 10.1093/ajcp/77.1.82

[4] Montoro-Huguet MA, Santolaria-Piedrafita S, Canamares-Orbis P, Garcia-Erce JA (2021) Iron deficiency in celiac disease: prevalence, health impact, and clinical management. Nutrients 13(10):3437 34684433 10.3390/nu13103437 PMC8537360

[5] Talarico V, Giancotti L, Mazza GA, Miniero R, Bertini M (2021) Iron deficiency anemia in celiac disease. Nutrients 13(5):1695 34067622 10.3390/nu13051695 PMC8156426

[6] Sanseviero MT, Mazza GA, Pullano MN, Oliveiro AC, Altomare F, Pedrelli L, Dattilo B, Miniero R, Meloni G, Giancotti L et al (2016) Iron deficiency anemia in newly diagnosed celiac disease in children. Minerva Pediatr 68(1):1–4 26864718

[7] Savilahti E, Kolho KL, Westerholm-Ormio M, Verkasalo M (2010) Clinics of coeliac disease in children in the 2000s. Acta Paediatr 99(7):1026–1030 20199495 10.1111/j.1651-2227.2010.01740.x

[8] Abdi F, Zuberi S, Blom JJ, Armstrong D, Pinto-Sanchez MI (2023) Nutritional considerations in celiac disease and non-celiac gluten/wheat sensitivity. Nutrients 15(6):1475 36986205 10.3390/nu15061475 PMC10058476

[9] Silvester JA, Weiten D, Graff LA, Walker JR, Duerksen DR (2016) Living gluten-free: adherence, knowledge, lifestyle adaptations and feelings towards a gluten-free diet. J Hum Nutr Diet 29(3):374–382 25891988 10.1111/jhn.12316

[10] Kassebaum NJ, Jasrasaria R, Naghavi M, Wulf SK, Johns N, Lozano R, Regan M, Weatherall D, Chou DP, Eisele TP et al (2014) A systematic analysis of global anemia burden from 1990 to 2010. Blood 123(5):615–624 24297872 10.1182/blood-2013-06-508325 PMC3907750

[11] Jefferds MED, Mei Z, Addo Y, Hamner HC, Perrine CG, Flores-Ayala R, Pfeiffer CM, Sharma AJ (2022) Iron deficiency in the United States: limitations in guidelines, data, and monitoring of disparities. Am J Public Health 112(S8):S826-s835 36288529 10.2105/AJPH.2022.306998 PMC9612197

[12] Mearin ML, Agardh D, Antunes H, Al-Toma A, Auricchio R, Castillejo G, Catassi C, Ciacci C, Discepolo V, Dolinsek J et al (2022) ESPGHAN position paper on management and follow-up of children and adolescents with celiac disease. J Pediatr Gastroenterol Nutr 75(3):369–386 35758521 10.1097/MPG.0000000000003540

[13] Cohen CT, Powers JM (2023) Intravenous iron therapy in pediatrics: who should get it and when is the right time? Hematol Am Soc Hematol Educ Program 2023(1):630–635 10.1182/hematology.2023000496 PMC10727076 38066925

[14] Saukkonen J, Kaukinen K, Koivisto AM, Maki M, Laurila K, Sievanen H, Collin P, Kurppa K (2017) Clinical characteristics and the dietary response in celiac disease patients presenting with or without anemia. J Clin Gastroenterol 51(5):412–416 27306936 10.1097/MCG.0000000000000556

[15] Sansotta N, Amirikian K, Guandalini S, Jericho H (2018) Celiac disease symptom resolution: effectiveness of the gluten-free diet. J Pediatr Gastroenterol Nutr 66(1):48–52 28514243 10.1097/MPG.0000000000001634

[16] Sari R, Yildirim B, Sevinc A, Buyukberber S (2000) Gluten-free diet improves iron-deficiency anaemia in patients with coeliac disease. J Health Popul Nutr 18(1):54–56 11014772

[17] Rozenberg O, Rinawi F, Haritan Y, Yerushalmi B, Kori M, Morgenstern S, Peleg S, Osyntsov L, Colodner R, Shamir R (2020) Automated analyzers are suited for diagnosing celiac disease without a biopsy. J Pediatr Gastroenterol Nutr 71(1):64–70 32265407 10.1097/MPG.0000000000002711

[18] Jericho H, Sansotta N, Guandalini S (2017) Extraintestinal manifestations of celiac disease: effectiveness of the gluten-free diet. J Pediatr Gastroenterol Nutr 65(1):75–79 28644353 10.1097/MPG.0000000000001420

[19] Burger JPW, van der Laan JJH, Jansen TA, Drenth JPH, Roovers EA, Wessels MMS, Wahab PJ (2018) Low yield for routine laboratory checks in follow-up of coeliac disease. J Gastrointestin Liver Dis 27(3):233–239 30240466 10.15403/jgld.2014.1121.273.jph

[20] Catal F, Topal E, Ermistekin H, Yildirim Acar N, Sinanoglu MS, Karabiber H, Selimoglu MA (2015) The hematologic manifestations of pediatric celiac disease at the time of diagnosis and efficiency of gluten-free diet. Turk J Med Sci 45(3):663–667 26281336

[21] Kivela L, Kaukinen K, Huhtala H, Lahdeaho ML, Maki M, Kurppa K (2017) At-risk screened children with celiac disease are comparable in disease severity and dietary adherence to those found because of clinical suspicion: a large cohort study. J Pediatr 183:115-121 e112 28153477 10.1016/j.jpeds.2016.12.077

[22] Rajalahti T, Repo M, Kivela L, Huhtala H, Maki M, Kaukinen K, Lindfors K, Kurppa K (2017) Anemia in pediatric celiac disease: association with clinical and histological features and response to gluten-free diet. J Pediatr Gastroenterol Nutr 64(1):e1–e6 27035377 10.1097/MPG.0000000000001221

[23] Assa A, Frenkel-Nir Y, Tzur D, Katz LH, Shamir R (2017) Large population study shows that adolescents with celiac disease have an increased risk of multiple autoimmune and nonautoimmune comorbidities. Acta Paediatr 106(6):967–972 28247429 10.1111/apa.13808

[24] Malaspina D, Corcoran C, Kleinhaus KR, Perrin MC, Fennig S, Nahon D, Friedlander Y, Harlap S (2008) Acute maternal stress in pregnancy and schizophrenia in offspring: a cohort prospective study. BMC Psychiatry 8:71 18717990 10.1186/1471-244X-8-71 PMC2546388

[25] Nestares T, Martin-Masot R, Labella A, Aparicio VA, Flor-Alemany M, Lopez-Frias M, Maldonado J (2020) Is a gluten-free diet enough to maintain correct micronutrients status in young patients with celiac disease? Nutrients 12(3):844 32245180 10.3390/nu12030844 PMC7146183

[26] Wessels MM (2016) van V, II, Vriezinga SL, Putter H, Rings EH, Mearin ML: Complementary serologic investigations in children with celiac disease is unnecessary during follow-up. J Pediatr 169:55–60 26547400 10.1016/j.jpeds.2015.09.078

[27] Radlovic N, Mladenovic M, Lekovic Z, Zivanovic D, Brdar R, Radlovic V, Ristic D, Pavlovic M, Stojsic Z, Vuletic B et al (2009) Effect of gluten-free diet on the growth and nutritional status of children with coeliac disease. Srp Arh Celok Lek 137(11–12):632–637 20069920 10.2298/sarh0912632r

[28] Canova C, Rosato I, Marsilio I, Valiante F, Zorzetto V, Cataudella G, D’Odorico A, Zingone F (2021) Quality of life and psychological disorders in coeliac disease: a prospective multicentre study. Nutrients 13(9):3233 34579108 10.3390/nu13093233 PMC8470791

[29] Silitonga HTH, Salim LA, Nurmala I, Wartiningsih M (2023) Compliance of iron supplementation and determinants among adolescent girls: a systematic review. Iran J Public Health 52(1):37–48 36824244 10.18502/ijph.v52i1.11664 PMC9941429

[30] Annibale B, Severi C, Chistolini A, Antonelli G, Lahner E, Marcheggiano A, Iannoni C, Monarca B, Delle Fave G (2001) Efficacy of gluten-free diet alone on recovery from iron deficiency anemia in adult celiac patients. Am J Gastroenterol 96(1):132–137 11197242 10.1111/j.1572-0241.2001.03463.x

[31] Stahlberg MR, Savilahti E, Siimes MA (1991) Iron deficiency in coeliac disease is mild and it is detected and corrected by gluten-free diet. Acta Paediatr Scand 80(2):190–193 2035309 10.1111/j.1651-2227.1991.tb11832.x

[32] Zriouel A, Cherkani-Hassani A, Khadmaoui A, Ettair S (2021) Evaluation of iron intake among adolescents with celiac disease in Morocco. Medico-Legal Update 21(1):1671–1676

